# Can immersive technologies rebuild heritage and sense of place? Examining virtual Reality’s role in fostering community resilience in post-disaster Italy

**DOI:** 10.1080/13527258.2025.2520760

**Published:** 2025-06-26

**Authors:** Paola Di Giuseppantonio Di Franco, Francesca Dolcetti, Matteo Baraldo, Steven Day

**Affiliations:** a School of Philosophical, Historical, and Interdisciplinary Studies – University of Essex, Colchester, UK; b ThinkSee3D, UK

**Keywords:** immersive technologies, community resilience, sense of place, Co-creation, lost heritage, Disasters

## Abstract

Disasters disrupt not only physical environments but also socio-cultural identities and sense of place. This study explores the role of Virtual Reality (VR) in post-disaster recovery, focusing on the earthquake-affected towns of Amatrice and Accumoli, Italy. Using a techno-ethnographic methodology, we integrated video interviews, 3D digital reconstruction, and co-creation to examine how immersive technologies facilitate emotional reconnection and community resilience. Findings suggest that VR environments can serve as spaces for mourning and re-familiarisation, helping individuals reconnect with lost surroundings. Multi-sensory elements (i.e. lighting, soundscapes, and everyday material details) proved crucial in fostering virtual place attachment. Intergenerational use of VR supported memory transmission, as younger community members relied on elders to interpret pre-disaster environments. However, trauma sensitivity remains essential, underscoring the value of community-centred, iterative design. This study contributes to digital heritage research by showing how VR can go beyond documentation, supporting storytelling and story-sharing, memory work, restauration of sense of place, and resilience-building, while calling for further study of its long-term impacts.

## Pro logue

How can we remove this panorama of destruction from our soul? ‘When I feel low, I go to Google Maps. The images of Amatrice have not been updated, so I can still walk through the old streets and see what was there before the earthquake. That day, we all somewhat died’. Resident of Amatrice, quoted in *Oggi*

(2022)

Another resident reflected: ‘We know the new town must be safer, but losing that aspect of the town hurts. I wish I had a miniature replica of Amatrice I could look at whenever I wanted’.

These reflections capture the emotional weight of disrupted place attachment and raise a central question: If outdated satellite imagery can offer mourning space, could immersive environments offer a deeper form of reconnection?

## Introduction

Italy, located at the intersection of the African and Eurasian tectonic plates, is among the most earthquake-prone regions in Europe. The 2016 Central Italy earthquake sequence caused catastrophic damage to historic towns such as Amatrice and Accumoli, with long-term consequences that extended beyond physical destruction. Heritage loss, displacement, and threat to community identity emerged as key challenges for recovery (Stewart et al. 2018).

In post-disaster contexts, the restoration of cultural heritage (both tangible and intangible) plays a critical role in re-establishing socio-cultural continuity, fostering a sense of place, and supporting community resilience. Traditional recovery efforts often emphasise physical reconstruction, yet such approaches can neglect the deeper emotional and cultural dimensions of loss. This paper argues that heritage-based recovery must include affective and social re-engagement, particularly through inclusive and community-centred approaches.

Recent scholarship in heritage studies has highlighted the need to incorporate digital technologies, not solely for documentation, but as active tools for community engagement, memory preservation, and community resilience (e.g. Jeffrey et al. 2020; Mason and Vavoula 2021). This study contributes to that growing body of work by examining how immersive Virtual Reality (VR), when co-designed with local communities, can be used to support emotional reconnection, intergenerational memory transmission, and collective reflection on heritage values in disaster-stricken areas.

Drawing on the case study of Amatrice and Accumoli, this research presents an applied, practice-based framework that integrates techno-ethnographic methods and co-designed 3D reconstruction. It explores the role of immersive technologies in reactivating a sense of place and in shaping new forms of cultural continuity after disruption. Through collaborative design processes and iterative feedback cycles, the project emphasises the ethical and affective dimensions of digital heritage practice.

The following questions guide this study:

How can immersive digital environments contribute to rebuilding a disrupted sense of place in post-disaster heritage contexts?

In what ways can co-created VR reconstructions support emotional recovery and community resilience?

How do local communities engage with digital heritage tools as part of a broader effort to preserve memory, identity, and cultural continuity?

This study aims to contribute to recent debates in critical heritage studies, particularly those exploring the emotional and affective dimensions of heritage engagement. Recent work by Smith and colleagues (Smith and Gary 2015; Smith, Wetherell, and Campbell 2018) highlights how emotion is constitutive of heritage practice (i.e. shaping how communities make meaning, respond to loss, and sustain cultural continuity). Emotional heritage is a process through which people negotiate identity, sense of belonging, and loss of heritage. In post-disaster contexts, such affective dynamics become especially crucial, as individuals experience grief, disorientation, and rupture to sense of place.

By situating this work within critical heritage studies, this paper positions VR as more than a representational tool; it is framed here as a socio-cultural platform for spatial and emotional reorientation, mourning, intergenerational storytelling and story-sharing, and place-making.

The case study of Amatrice and Accumoli offers broader insights for heritage professionals and policymakers working at the intersection of disaster recovery, digital innovation, and community engagement.

## Socio-cultural role of 3D technologies in post-disaster contexts

This section reviews the key theoretical foundations of sense of place and community resilience, followed by an exploration of how 3D and VR technologies are being applied in disaster recovery contexts. It highlights both their technical affordances and socio-cultural impacts, identifies gaps in current research, and positions this study within these broader debates. While the technical advantages of 3D technologies, such as simulations and situational awareness (Esposito et al. 2022; Kedia et al. 2022; Khan, Gupta, and Kumar Gupta 2020), have been extensively documented, this paper focuses on their socio-cultural potential for rebuilding disrupted heritage and fostering community recovery.

In the context of post-disaster recovery, sense of place and community resilience are critical interconnected concepts that are essential for helping communities recover and maintain their sense of identity and social cohesion after a disaster. Sense of place refers to the connection individuals and communities develop with physical spaces. It encompasses both tangible elements (i.e. monuments, architectural and environmental landmarks) and intangible associations, including shared social practices, rituals, and emotional bonds tied to place (Foote and Maoz 2009). Place is not merely spatial, but also a social and political construct embedded in collective memory and shaped by acts of remembering and forgetting. The work of geographer Tuan (1974; 1977) offers a foundational distinction between space and place and conceptualises place as a lived, remembered, and emotional experience. His notion of *topophilia*, the love of place, is especially relevant to post-disaster contexts, where loss and belonging shape community identity. Complementing this, geographer Relph (1976, 55) explores how people experience *existential insideness*, that is, a sense of being at home in place, and how this can be disrupted by trauma or erasure. These theoretical perspectives help frame why the emotional and cultural dimensions of heritage are indispensable in recovery processes, even as they are frequently overlooked by disaster recovery frameworks (Crowley et al. 2022; Jigyasu et al. 2013).

Following the 2016 earthquake that devastated Amatrice and Accumoli, many residents reported a disorientation that went beyond physical displacement. The concept of ‘interrupted landscapes’ (Clemente and Salvati 2017) captures this rupture in spatial and cultural continuity. When familiar environments are altered or destroyed, everyday routines, social connections, and sensory cues are lost, eroding the foundations of individual and collective identity.

In response, scholars have proposed frameworks that link emotional dislocation with social resilience-building. Cox and Perry (2011) model of disorientation and reorientation emphasises the importance of place in post-disaster recovery, not simply as physical infrastructure, but as a cognitive, social, and cultural reference point. They argue that reorienting oneself in space is central to reconstructing social capital and identity. Similarly, Scannell et al. (2016) note that place attachment is both emotional and functional, shaping decisions about whether to return and reinvest in community life. Rebuilding place, then, is not just a material process; it is a social, cultural, and emotional one.

The concept of community resilience, often defined as the capacity to adapt, reorganise, and maintain cohesion following crisis, has also evolved. Earlier paradigms focused on ‘bouncing back’ to pre-disaster conditions, but this approach has been critiqued as insufficient and at times counterproductive (Chandler and Jon 2017; Manyena 2006). The Sendai Framework for Disaster Risk Reduction (United Nations 2015) reframes resilience as the ability to ‘bounce forward’, that is, to rebuild in ways that address vulnerability, foster innovation, and sustain cultural continuity. This shift emphasises the need for heritage recovery strategies that support both physical adaptation and socio-cultural regeneration (Binder, Baker, and Barile 2015; Haque and Etkin 2007).

Together, these perspectives suggest that reclaiming sense of place is central to resilience-building. In this context, digital tools such as VR, photogrammetry, and 3D scanning can offer new ways of engaging with disrupted heritage. While much attention has focused on their technical capacities (i.e. for spatial simulation, decision-making, or training; Esposito et al. 2022; Kedia et al. 2022; Khan, Gupta, and Kumar Gupta 2020), less work has explored their emotional and socio-cultural affordances.

Virtual Reality, is particularly promising for post-disaster heritage recovery, because it enables an experiential *re-inhabiting* of lost or inaccessible places. As Relph (2007) argues, virtual realities have the potential to evoke a *spirit of place* when they enable users to personally identify with a digital environment, recall meaningful narratives, and engage their senses. In his view, for a virtual place to be perceived as ‘authentic’, it must not only mimic physical realism, but also convey memory, emotional familiarity, and a sense of identity and belonging. This aligns with Mel Slater’s concept of *place illusion* (2009), that is defined as the phenomenon by which users feel physically present in a virtual space, even when the users know they are not. This element suggests that VR can create affective experiences that help reconstitute disrupted place attachment. This potential is beginning to be realised in a range of digital heritage initiatives, some of which rely on 3D reconstruction techniques to document and preserve damaged cultural sites.

Digital documentation in disaster contexts often involves crowdsourcing for 3D reconstructions of cultural heritage. A notable example is *Rekrei* (former *Project Mosul*; endnote 1), a global project that uses crowdsourced 3D reconstructions to digitally preserve heritage sites destroyed by conflict or disaster. While *Rekrei* demonstrates the power of collective memory and a collaborative effort in reconstructing lost heritage, some scholars, including Vincent et al. (2015), highlight challenges such as sustaining long-term participation and ensuring inclusivity in these projects.

Other heritage-focused projects illustrate how participatory approaches can bridge gaps between technological tools and socio-cultural recovery. Devilat et al. (2024) work in Gujarat, India, illustrates how combining 3D scanning with ethnographic methods fosters a shared sense of ownership over reconstruction efforts. By engaging local communities through large-scale visualisations and interactive tools, the project not only documented heritage but also facilitated dialogue and collective memory building, key aspects of restoring a sense of place. This initiative used large-scale aerial maps laid on the floor and perspective sections of interior spaces to foster community dialogue. Additionally, 3D representations and architectural projections were compiled into an accessible booklet for inclusive engagement.

Similarly, Dionisio et al. (2016) work with Māori communities shows how 3D visualisation tools can democratise planning by integrating indigenous perspectives into post-earthquake rebuilding processes. The study employed Envision Scenario Planning (ESP), a 3D visualisation system for planning, to assist communities in post-disaster decision-making and assess its potential to enhance community engagement in reconstruction processes. This research aligns with the principles of Public Participation Geographic Information Systems (PPGIS), which aim to democratise GIS technologies (Kwaku Kyem Peter 2000). Similarly, Zhang et al. (2017) examine the design of a Disaster Prevention Park using a cloud-based 3D platform. This platform enables stakeholders to propose design concepts and discuss design alternatives during online design review meetings. Researchers say 3D enhances visualisation and co-design, fostering inclusive dialogue and supporting equitable, collaborative rebuilding. When it comes to VR, there is limited published research leveraging the immersive capabilities of VR in disaster management to create meaningful social impact. When compared with VR, 3D technologies may be better at overcoming hardware limitations for real-time rendering of spaces – particularly large ones – thereby reducing the technological and digital divide and lowering barriers to adoption in real-world settings. However, despite these limitations, VR offers affordances that go beyond accurate reproduction. Its immersive and affective dimensions allow users to re-engage with lost or inaccessible places in emotionally resonant ways. For example, Rozhen and Mohammed (2022) discusses the use of VR to recreate sites of war and violence, through the example of the VR and AR exhibition of the Yazidi genocide, offering a digital space for mourning, and reflection. The project foregrounds affective engagement, allowing users to re-enter meaningful cultural environments through immersive interaction. VR artistic experiences are also relevant in rebuilding a sense of place and enhancing community resilience. *The World Came Flooding In* (endnote 2) by Isobel Knowles and Van Sowerwine exemplifies how VR storytelling evokes emotional connections to lost places, offering a model for integrating artistic practices into post-disaster recovery frameworks. This VR experience supports individuals coping with trauma from flooding using cardboard objects recreated by local communities that are transformed into a virtual environment (Andrews et al. 2024).

While these studies highlight the potential of 3D and VR technologies in disaster recovery, few address their role in restoring a disrupted sense of place or fostering community resilience.

The research presented in this paper bridges this gap by examining how community-centred 3D applications can support both emotional and cultural recovery in post-disaster contexts. By reconnecting individuals with lost environments, this approach aims to rebuild a disrupted sense of place while enhancing community cohesion, thereby addressing not only the technical challenges of digital reconstruction but also the socio-cultural dimensions essential for sustainable disaster recovery. The following section details the techno-ethnographic methods employed in the REPLACE project, which combines ethnography and co-creation of 3D immersive VR design and development to explore these themes.

## Creation and development of VR applications in Amatrice and Accumoli

### Methods

This study was conducted as part of the UKRI-funded REPLACE project, which explores how immersive technologies can support heritage-based recovery and community resilience in disaster-affected areas. The research design employed a techno-ethnographic methodology, combining ethnographic fieldwork with the design, development, and assessment of digital heritage tools – specifically, VR reconstructions of pre-earthquake environments in Amatrice and Accumoli, Italy.

This approach builds on emerging practices in critical heritage studies and participatory design (Christensen 2014; Dolcetti 2024; Wellner, Botin, and Otrel-Cass 2015), aiming to create ethically grounded digital heritage experiences that foster affective engagement. REPLACE’s techno-ethnography is structured around four interconnected pillars:

*Ethnography*: Ethnographic methods included walking interviews, focus groups, and co-design workshops to document memory, heritage values, and emotional ties to lost places – ensuring that VR reconstructions captured not only architectural accuracy but also cultural and affective resonance. Participants were selected through purposive and snowball sampling to ensure diversity in age, gender, socio-economic background, and social roles (e.g. council members, business owners, retirees, students). This strategy mitigated dominant voices and captured a range of perspectives relevant to post-disaster recovery. A total of 26 participants (aged 7–80; 14 female, 12 male) took part in in-depth interviews. They included both residents and non-residents (many second-home owners with inherited properties), who had experienced the earthquake directly or were born shortly before or after it. Community partners (Radici Accumolesi in Accumoli and Casa delle Donne in Amatrice) facilitated follow-up workshops and feedback sessions.

*Digital Data Collection*: 3D modelling was developed using photogrammetry, drone imagery, and GIS data. Technical workflows prioritised cultural fidelity over visual spectacle, with community preferences guiding site selection and detailing.

*Digital Experience Design*: Reconstructions were developed using Unreal Engine, integrating terrain modelling, lighting simulation (Ultra Dynamic Sky), texturing (PBR materials, field photography), and interactive affordances. Environmental soundscapes and physical cues (e.g. seating, bells) were added to enhance sensory engagement.

*Evaluation and Feedback*: Community testing sessions were video-recorded and analysed through Thematic Analysis (Braun and Clarke 2006) using NVivo. Feedback informed iterative refinements. Data were anonymised and analysed using inductive and deductive coding, combining research questions with emergent themes. Before each session, participants are asked to sign the consent forms approved by the University of Essex Ethics Committee. Participants are also asked to complete a personal data survey. Results from the qualitative analysis are anonymised by assigning participants pseudonyms (Moore 2012).

**Figure 1.**
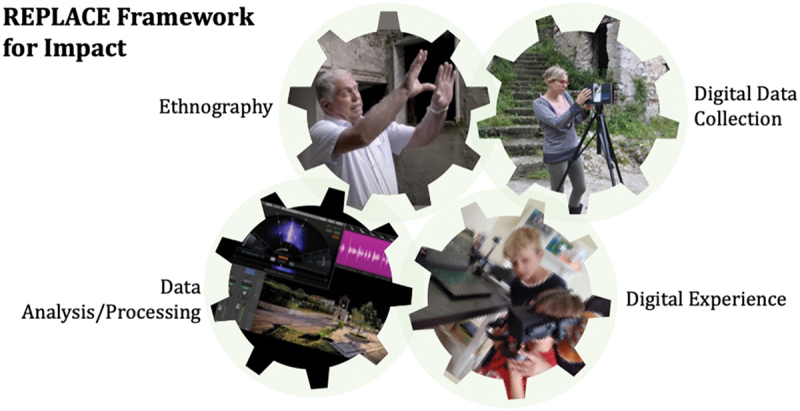
REPLACE’s techno-ethnography.

**Figure 2.**
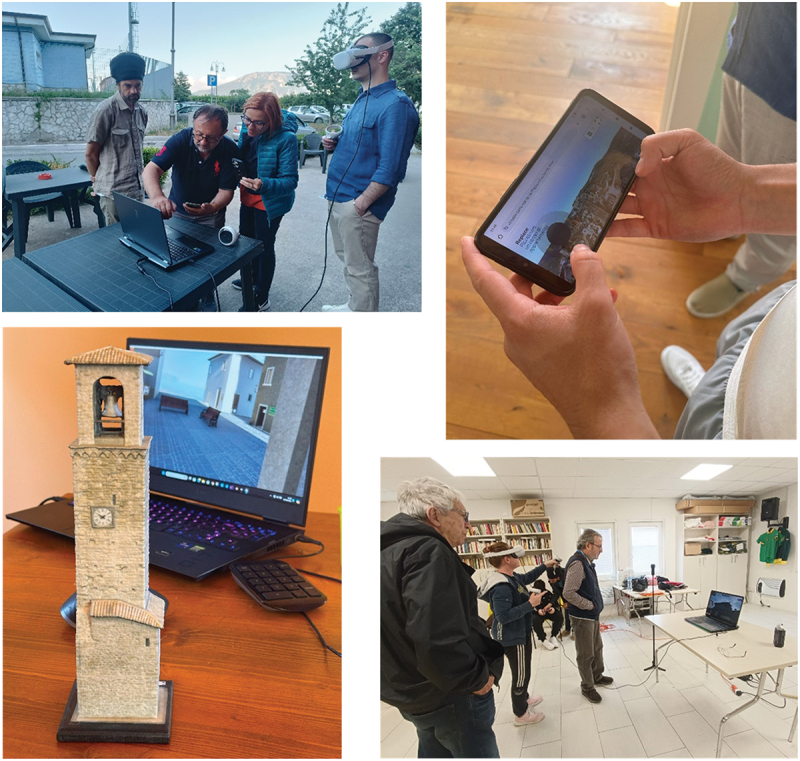
Images showcasing community events where participants engage with and evaluate 3D digital content using various interactive technologies.

This iterative process ensures that the community’s cultural, emotional, and social needs are integrated into the development of immersive VR environments, fostering both emotional recovery and a restored sense of place (Figure 1). During this collaborative process, researchers and 3D content creators worked closely with community members and stakeholders to develop immersive VR experiences that reflect the cultural and emotional significance of their lost places. Although the development of VR immersive experiences required specialist expertise, communities engaged with REPLACE on a spectrum of participation, ranging from consultation to collaboration (Cooper et al. 2021; Gonzàlez 2020). This approach maintained a balance between community input and the technical demands of VR creation (Figure 2).

Despite careful planning, the development and deployment of VR environments presented notable technical challenges. The resource-intensive nature of immersive reconstructions increased the risk of performance issues and instability, particularly during community demonstrations. Beta versions of the VR application required frequent testing and debugging, and unexpected system failures occasionally disrupted sessions – sometimes necessitating full device restarts. In community settings, these issues risked undermining participants’ confidence in the technology and the reliability of the intervention. These challenges highlight the complexity of integrating community feedback with high-performance digital heritage tools in an iterative and co-designed process.

### Research findings. Effects of VR applications on community resilience and sense of place

In the earthquake-stricken areas of Accumoli and Amatrice, where reconstruction has only recently begun and many historic centres remain inaccessible, VR technologies emerged as affective tools for re-engaging residents with their disrupted environments. Participants frequently described the VR experiences as emotionally intense and deeply meaningful, often articulating strong responses linked to memory, grief, and identity. This section presents five key thematic insights drawn from participant narratives, focusing on how immersive digital heritage interventions support sense of place and community resilience in post-disaster contexts.

### Grief and mourning through VR

Findings indicate that VR served as both a space for grief processing and a tool for reconnecting with lost places, reinforcing participants’ emotional attachment to their pre-disaster environments. Many participants reflected on how the digital replica could be a space where they could mourn and reflect on the temporary or permanent loss of the cherished environment. One participant noted that the experience felt like ‘a cemetery of place’, emphasising that digital spaces can serve memorial functions similar to ritual sites in the physical world.

Participants engaged with the VR environments not only to observe familiar structures but to emotionally revisit lost routines and attachments. Giada, for instance, described her desire to revisit San Francesco Square, associating it with memories of lost family members. Others, like Alberto, became emotionally overwhelmed and had to pause the experience. These responses illustrate the potential of VR to provide therapeutic landscapes, where grief is not erased but confronted in affective, community-centred ways.

However, the VR experience was not always positive. Some participants struggled with the trauma of reliving destruction. When we presented a first prototype of the VR reconstruction of Accumoli in May 2024, the immersive experience mainly showed the town in the actual state of ruins, apart from the reconstruction of a small portion of the historic centre, namely a cafe and few shops near the San Pietro Gate. We decided to present the VR experience in this early stage to collect feedback and ensure that the on-going modelling process would focus on reconstructing places of significance for community members. During this event, when Serena, a non-resident visitor, explored a night-time VR scenario, she became disoriented and distressed, as she could not recognise familiar landmarks or natural elements. She stated, ‘Is this Accumoli after the earthquake? Are these trees?’ but soon became increasingly uneasy and frightened, ‘Oh my god, how eerie! I see all these dark things around me’, to the point that she abruptly interrupted the VR exploration. Her reaction highlights the importance of trauma-informed design that can accommodate individual and collective sensitivities. In response, the research team adjusted lighting and environmental settings, allowing people to modify their VR experience. This shows the importance of iterative, community-centred and emotionally sensitive design for technological development.

### Reclaiming sense of place

A central theme in the study was how the VR facilitated re-familiarisation with the damaged landscapes, addressing participants’ disorientation caused by the devastated environment and forced displacement. Many residents live on the outskirts of their towns, forced away from the historic centre that was once the heart of their personal, professional, and social lives. For others (including the second-home owners), these historic centres were vibrant social hubs – the reason to return regularly and maintain a connection with their town (especially to spend their holidays during the summer). The daily view of these now inaccessible places reduced to a mix of void and debris is painful and both emotionally and physically disorienting, making it challenging to reconcile memories with the present reality: ‘I do not have the courage to go to Amatrice, because I would have to drive through the main road and see the destruction. It is like reliving the grief daily’ (Sandra, a resident of Accumoli).

By contrast, the VR offered participants a way to revisit the familiarity of what are now altered environments, regaining a sense of orientation and spatial familiarity, and to make sense of what the lost place signified for their self- and community identity. This aligns with the idea that reclaiming a disrupted landscape is integral to rebuilding community identity (Santangelo et al. 2022).

**Figure 3.**
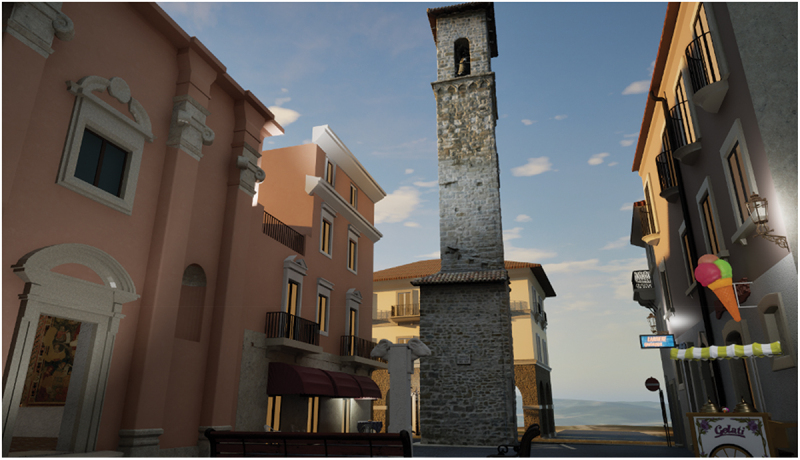
A view of a reconstructed Piazza San Giovanni in Amatrice, including the 3D-scanned civic tower.

Participants defined sense of place as an interplay between iconic landmarks and the smaller, multi-sensory details of everyday life. For example, Alberto, a resident of Accumoli, became overwhelmed with emotion during his virtual exploration of the town’s historic centre, later describing his joy at being able to roam the streets of Accumoli again (endnote 3). Similarly, Filomena (a resident of Amatrice) expressed her desire to ‘remain there’ while engaging with the VR reconstruction of Amatrice’s main square (San Giovanni): ‘I want to stay here! I want to stay here and eat an ice cream while sitting on the San Giovanni church’s steps and then drink some water from the fountain’ (endnote 4, Figure 3).

For most participants, virtually ‘returning’ to what used to be one of the town’s main social hubs brought back happy memories of community life and gatherings, especially during summer nights when people used to go out for a stroll in the town centre after dinner. As the physical place has been lost, so have the social dynamics associated with it and the community has been unable to recreate them elsewhere:

In the summer, we used to go out for a walk in San Giovanni after dinner almost every night, it was like our living room [meant as the heart of the home’s social life]. When my children were asking me where I was headed, I always replied ‘I am going to the living room!’ (Maria, resident of Amatrice)

**Figure 4.**
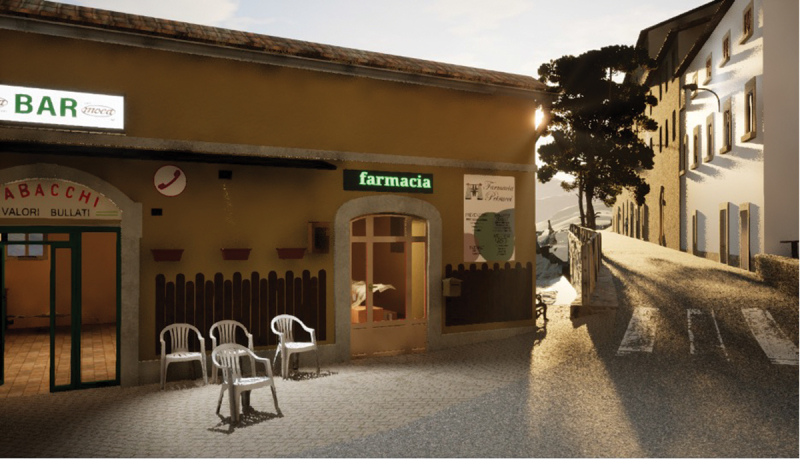
Accumoli’s reconstructed digital bar (i.e. Eng.: *café*) during a digital sunset.

The personal and collective significance placed upon the lost places particularly emerged when we asked participants how they wanted to implement the VR reconstruction and what they felt was important to include. On several occasions, participants began exploring the VR by looking for their homes and expressed a desire to include them in the reconstruction. Some participants also mentioned specific features in the communal areas, such as the flowerpots and trees that were adorning San Giovanni or the plastic chairs outside one of the cafes in Accumoli’s town centre (Figure 4), highlighting the importance of these details for reconnecting with their lost places. When showing our digital interventions at a community event in September 2024, a member of the REPLACE team invited Giorgia (a resident of Accumoli) to sit on a chair, to have a more embodied experience of the cafe’s VR reconstruction. Integrating a physical element evoked memories of familiar moments, such as hanging out at the cafe, sitting outside, and having a drink while ‘smoking many, many cigarettes!’ Similarly, others stated they wanted to hear the civic tower bells ringing: ‘before, the bells used to ring every hour, now we only hear them on special occasions such as the patron saint’s day or the anniversary of the earthquake’ (Maria, resident of Amatrice); or the chatter of people hanging out in San Giovanni’s square. Lucrezia movingly noted the absence of one crucial element, the smell of Amatrice’s town centre: ‘every street and alley had its own characteristic scent; I am not sure how to describe it’ (endnote 5).

**Figure 5.**
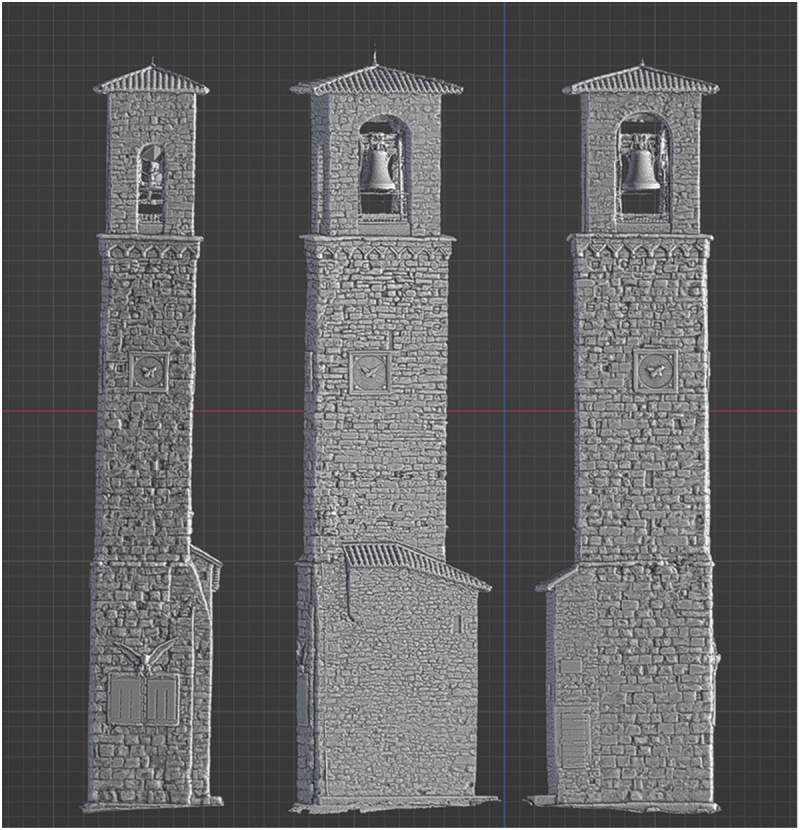
Amatrice civic tower (detailed 3D model from drone scan).

These emotional and sensory connections reveal how VR can go beyond visual accuracy and fidelity to evoke a sense of place by restoring both tangible and intangible dimensions of heritage. However, the subjective nature of memory emerged during interactions with the reconstructions. For instance, the steps of Sant’Antonio, a deconsecrated church used as a storage facility in San Giovanni square, became a focal point for memory recollection. During the VR demonstration, several residents asked for the steps to be included in the reconstruction. The developer initially referred to a photograph of the square for accuracy, which depicted several steps. However, one participant, recalling the emotional significance of this location, argued that the steps were larger and more numerous, explaining, ‘We used to sit there to chat and enjoy gelato from the nearby shop’. Another debate arose over the number of clocks on Amatrice’s civic tower, the city’s only surviving landmark, with some participants recalling one clock and others recalling four, even though a photograph and drone data confirmed there were three (Figure 5). These discrepancies underscore how personal experiences and cultural expectations mediate memory; due to the reconstruction works, people can only see the tower from the side road instead of walking around it as they used to. As Maria stated, when they walk on the road alongside the city centre, they often struggle to remember how it looked ‘because there is nothing left there, and we are starting to forget’. These collective experiences reveal how the interplay between digitally reconstructing tangible aspects of a lost place and intangible elements such as sensory cues and collective memories is what allows VR to capture a sense of place.

### Inter-generational dialogues

Our research findings indicate that VR reconstructions can contribute to community resilience by fostering intergenerational memory-sharing and dialogues about place and sense of place. Younger participants, who were too young to fully remember their towns before the earthquake, often relied on guidance from older family members to navigate and understand the VR environments. For instance, Lucia, a resident of Amatrice who was 9 years old in 2016, said she has only a vague memory of the city centre and the family house at the corner of the main square; when experiencing the VR reconstruction, she asked her mother for guidance in locating the window of her childhood bedroom, as she could not remember where it was. Similarly, Nicholas, a young resident of Accumoli who was only 3 years old at the time of the earthquake, found the VR environment unfamiliar and repeatedly sought help from his parents to navigate it.

This interactive process transformed the VR sessions into a communal storytelling experience, where one person would wear the headset, and other people would gather around the laptop to observe and comment on the VR experience in real time. In fact, while the current setup of REPLACE’s VR reconstructions is designed for individual immersive experiences, participants very rarely engaged with the VR in isolation. This dynamic not only enabled younger participants to learn about the town’s cultural history but also encouraged older generations to reflect on and share their memories, reinforcing a sense of continuity and belonging. As Francesco, a resident of Amatrice, remarked, although his son was born two years after the earthquake, he has ‘seen’ Amatrice as it was before the earthquake through the eyes of his parents and family members.

Luisa expressed a clear appreciation for the REPLACE project. She mentioned how by offering the opportunity ‘to travel back in time’ to a place that was part of their daily lives, the VR experience can support the community in keeping the memory alive:

[…] We realized that over time, many people are losing their memories, their points of reference, and even the smallest details are starting to fade. So, we seized the opportunity offered by this [REPLACE] project to search for fixed memories, situations, and perhaps even future perspectives. We wanted to listen to and engage with the community to try to understand what they still remember and what they would like to see. [in the future]

These dynamics reveal that VR technologies can support not only post-disaster healing, but also the intergenerational continuity of cultural knowledge.

### Feeling regarding realism and perceived ‘authenticity’

Another theme identified was the complex relationship between realism and perceived ‘authenticity’ in VR reconstructions. Building on the previous themes analysed, participants’ feedback on the VR environment revealed how perceptions of realism and perceived ‘authenticity’ were closely linked to their personal and collective memories of the place. The reactions to the VR environment emphasised the importance of small details and sensory elements in recreating a sense of familiarity and connection to the VR environment. For many, the VR experience evoked a sense of uncanniness (endnote 6). Although initially disorienting, this sense of uncanniness ultimately generated positive emotions. Beatrice, a resident of Amatrice, exclaimed, ‘Oh my God, how disturbing!’ No, I mean disturbing in a good way, I swear! How cool, so beautiful’. For Luisa, the feeling of uncanniness came from the ‘strange feeling’ of standing virtually in the middle of San Giovanni Square and looking up at the civic tower, as she used to do almost every day (endnote 7).

**Figure 6.**
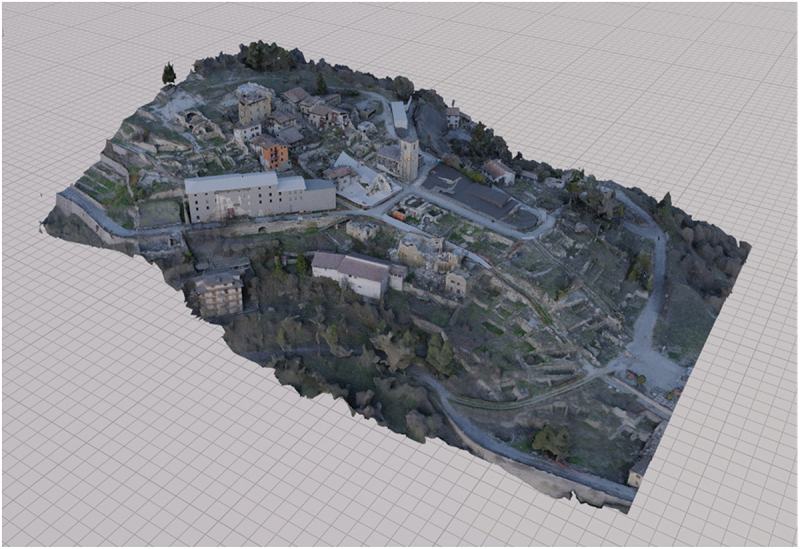
Photogrammetry model of Accumoli’s ruins with some reconstructed buildings.

**Figure 7.**
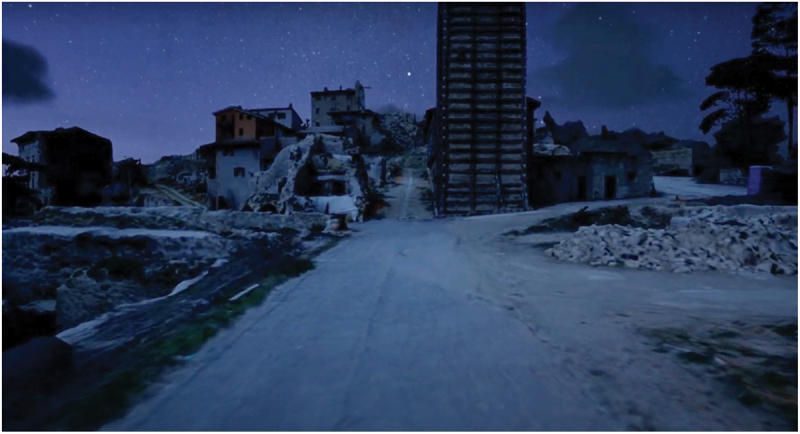
A view of Accumoli’s VR reconstruction first prototype.

Conversely, when experiencing the first prototype of Accumoli’s VR reconstruction, which mostly presented the current state of destruction, she felt entirely different; it felt ‘more real’ she said (Figures 6,7). When a researcher asked why, she explained that

unfortunately, this is our everyday reality now. If you had not told me, I would not have known that this is Accumoli; it could have been any of the nearby hamlets, because the houses are half destroyed, the road is still closed, and there is debris all around. It is a situation still suspended; it does not show any sign of reconstruction.

Her words suggest an interesting paradox: while the VR reconstruction of Accumoli’s devastated landscape was highly accurate, this realism seemed to blur the town’s distinct characteristics, making it feel indistinguishable from other earthquake-stricken towns. This highlights a crucial aspect of sense of place: in both reality and VR, it is not defined by physical accuracy alone but by the emotional ties (i.e. intangible heritage) linked to specific material and architectural elements (tangible heritage) that comprise memory and identity. In other words, a place’s meaning is shaped not just by how it looks in the present but by how it is remembered and emotionally experienced. Moreover, her response suggests that while she recognises the accuracy of the ruined landscape, she cannot characterise it as *her* place. Instead, the destruction renders it unfamiliar. Despite this being the reality of Accumoli today, she feels disconnected from it, and this seems to emphasise how devastation can really destroy the identity of a place and undermine a sense of place.^1^

Participants frequently associated realism and perceived ‘authenticity’ with the inclusion of small, seemingly mundane details that embodied their daily lives before the earthquake. Guido, also from Amatrice, noted that the future rebuilt town might probably resemble the ‘cleaner, more modern version of Amatrice’ depicted in our simulation. He compared this to the appearance of the city of L’Aquila after its reconstruction, following the earthquake that devastated the city in 2009. This sentiment was shared by other participants, who often remarked that the VR reconstruction’s spotless aesthetics felt uncanny, suggesting it should include dirt, wear, and other elements of daily activity to enhance what they described as the ‘authenticity’ of the town. Soundscape further influenced perceptions of realism and perceived ‘authenticity’. During a VR demonstration, Ginevra, a resident of Accumoli, while strolling through the virtual streets, exclaimed, ‘Oh, there is a party going on here!’ This impression was prompted by a town band member playing traditional music just outside the demo room. This accidental soundscape not only added an unexpected layer of cultural familiarity (reflecting intangible elements that resonated deeply with the community of Accumoli) but also seemed to enhance Ginevra’s sense of place in the VR space. Her reaction suggested excitement, as if she had momentarily found herself back in a familiar communal gathering.^2^

### Adaptation to technology and engagement

Despite initial concerns about digital unfamiliarity, most participants adapted quickly to the VR experience. After short tutorials, people of diverse ages and backgrounds explored the environments comfortably, often expressing excitement at interactive features. Rather than alienating people, the technology was received with curiosity and enthusiasm, reinforcing the accessibility of immersive heritage tools.

Only one participant discontinued the session due to motion sickness. Overall, participants described the experience as engaging, meaningful, and socially enriching. The findings suggest that with minimal onboarding, immersive technologies can be integrated effectively into community heritage contexts, bridging generational divides and supporting inclusive engagement.^3^

## Discussion

This discussion reflects on the key themes emerging from the findings and situates them within broader debates in digital heritage, post-disaster recovery, and immersive technologies. The analysis demonstrates how Virtual Reality can function not only as a tool for documentation but as an affective medium for memory transmission, mourning, and community resilience-building. Three core themes are addressed below: the role of VR in restoring sense of place and emotional recovery; the value of co-creation and participatory heritage practices; and the complex interplay between realism, memory, and authenticity in post-disaster digital environments.

### Restoring sense of place and facilitating emotional recovery

This study confirms that sense of place, recognised as foundational to community identity and post-disaster recovery (Cox and Perry 2011; Scannell et al. 2016), can be partially reactivated through immersive digital environments. The VR reconstructions developed through REPLACE offered participants an emotionally resonant way to re-engage with spaces that had been lost or rendered unrecognisable. The spatial immersion afforded by VR helped participants navigate memories, grieve for the loss, and re-establish cultural and emotional attachment to familiar places.^4^

These findings reinforce the idea that immersive technologies can become emotional infrastructures, spaces not only to see but to feel, reflect, and process loss (Galeazzi et al. 2023; Jeffrey et al. 2020). Similar to the work of Andrews et al. (2024), this study reinforces that digital reconstructions can support emotional recovery when designed with care, sensitivity, and community input.

However, the emotional intensity experienced by some participants also suggests the necessity for trauma-informed design. The ability to control visual and ambient features, adjust lighting, or pause the experience proved essential in mitigating distress. These findings align with emerging calls in digital heritage and immersive media for people agency and emotional adaptability (Abdalla 2024).^5^

### Co-creation and community involvement

Our study also highlights the importance of community engagement in all stages of the design process. While VR has been widely used for post-disaster digital reconstructions of lost heritage, many projects remain top-down, expert-driven initiatives that focus primarily on technical accuracy and a standardised definition of ‘authenticity’, rather than community-centred design (Baraldo and Di Giuseppantonio Di Franco 2024; Di Franco, Paola, and Vassallo 2018; Dolcetti 2024). The iterative co-creation element of the project certainly fostered collective experiences, but also (and especially) inter-generational dialogues. Younger participants relied on older family members to guide them through virtual reconstructions, bridging generational gaps and ensuring the continuity of cultural knowledge and memory of the place. Shared VR experiences, where family and friends observed and commented in real time, turned individual exploration into communal storytelling. This finding suggests how VR can play a role in building community cohesion in these post-disaster contexts by creating spaces for shared reflections and dialogue.

Despite the continuous and iterative engagement with communities, REPLACE, like similar projects discussed in the literature review, faces barriers to achieving fully participatory design due to the technical complexity of the digital reconstruction process. This limitation is partially addressed through iterative VR development cycles that incorporate ongoing community feedback.^6^

### Tensions between realism, perceived ‘authenticity’, and memory in digital post-disaster reconstruction of heritage and place

Participants frequently emphasised the importance of realism in VR environments, from small details, like flowerpots and church steps, to sensory elements like sounds or smells. This attention to details enhances the perceived realism of the reconstruction and fosters deeper emotional connections. As mentioned above, the VR experience was often a collective experience with one person wearing the VR headset and friends and/or relatives looking at the 3D reconstructions experienced by that person through a computer screen. Shared memories about the details of specific landmarks often enhanced communal discussions. Discrepancies between participant memory and archival evidence, such as debates about the number of clocks on the civic tower, reinforce the idea that memory is subjective and emotionally mediated and suggest how the search for realism in VR plays a role in reinforcing community identity and collective memories.^7^

## Conclusion and recommendations

This paper has explored how immersive VR environments, when developed through co-design and ethically sensitive methods, can contribute to heritage recovery and community resilience in post-disaster contexts. Drawing on ethnographic fieldwork and iterative co-creation with communities in Amatrice and Accumoli, the study has demonstrated that digital heritage tools can serve not only as documentation platforms, but as affective and dialogic spaces where memory, mourning, and sense of place are actively reconstructed.

Our findings show that VR extends beyond documentation and planning for tangible heritage preservation, offering opportunities for emotional engagement, community reconnection, and disaster management. By enabling people to interact with virtual reconstructions of lost environments, VR fosters memory recollection, intergenerational dialogue, and knowledge transfer.

Yet, long-term sustainability remains a key challenge. Digital heritage projects often face technological obsolescence and short-term funding constraints, risking the loss of access as hardware evolves or grants expire (Baraldo and Di Giuseppantonio Di Franco 2024). As part of this project’s sustainability strategy, reconstructions will be hosted on the digital infrastructure of the Italian National Council of Research (CNR), the H2IOSC initiative (endnote 8), ensuring durable access for communities and researchers (Fanini 2021). This will also enable us to add Augmented Reality elements in the reconstructed towns, when the reconstruction will be completed in Accumoli and Amatrice.^8^

Future research should explore the integration of additional sensory modalities (i.e. such as smell and haptic feedback) to enhance immersion. Expanding VR applications across diverse disaster-affected contexts would also help evaluate their global relevance and adaptability. Longitudinal studies are needed to assess the sustained impact of VR on emotional recovery and community resilience. Moreover, investigating how younger generations without direct pre-disaster memories will use VR to construct a sense of place offers an important line of research.

This study also highlights key considerations for the design and implementation of future digital heritage interventions. Effective practices must centre on iterative, community-driven development to ensure cultural relevance, ethical integrity, and equitable access. A community-centred approach fosters sustainability while enabling cultural landscapes to be reconstructed according to local values, memories, and priorities.

The success of VR in post-disaster heritage work depends not only on technical precision but on its responsiveness to evolving community needs, both in the short term (emotional and cultural reconnection) and long term (resilience-building). Critical strategies should include modular system design for easy updates, the use of open-source platforms to support preservation and ownership, and integration into broader Disaster Risk Reduction (DRR) frameworks. Embedding these tools into DRR protocols requires collaboration with local authorities, cultural heritage institutions, NGOs, and policymakers to ensure that VR becomes a practical component of recovery and preparedness plans.

To conclude, while our study suggests how VR can be an important tool for fostering memory recollection, grief processing, and intergenerational dialogue, as well as enhancing sense of place and increasing community resilience, we must recognise its limitations and reject the assumption that digital solutions alone can resolve complex socio-cultural recovery processes. Post-disaster recovery is multifaceted and involves emotional, economic, social, cultural, and political dimensions that cannot certainly be addressed by technology alone. For us, VR is not treated as a replacement for other heritage practices (especially grassroots heritage practices put in place by the community itself), but as a complementary tool that enhances existing forms of identity building, memory recollection, and heritage practices that aim to foster people’s reconnection with the damaged and/or lost place. By taking these considerations into account, we position digital heritage interventions not as isolated technological fixes but as part of a broader system of community-centred approaches for disaster recovery. This perspective ensures that our research remains socio-culturally and ethically responsible.

## Acknowledgments

We sincerely thank the citizens of Amatrice and Accumoli for their invaluable participation in this research. Special appreciation goes to *Radici Accumolesi*, *Casa delle Donne in Amatrice*, and the municipality of Senerchia, particularly Concetta Varalla, Mino DeVita, and Carmine Sessa, for their support and collaboration. We also extend our gratitude to Louise Rodwell for her comments to the paper and Catrina Appleby for her final copyediting. This work was generously supported by the UKRI (UK Research and Innovation) FLF (Future Leader Fellowship) grant number MR/W009153/1 and is part of a four-year research programme titled ‘Rebuilding a Sense of Place (REPLACE): The Socio-cultural Role of 3D technologies in Increasing Community Resilience after “Natural” Disasters’.

## Notes

1. https://rekrei.org/ (Accessed on 24 February 2025).

2. https://www.isobelandvan.com/work/theworldcamefloodingin (Accessed on 24 February 2025).

3. https://youtu.be/CYOAZALq4W8?feature=shared. (Accessed on 2 June 2025).

4. https://www.youtube.com/shorts/fdNqvSMkiRE(Accessed on 2 June 2025).

5. https://youtube.com/shorts/x-Bo1QFvOGg?feature=share (Accessed on 2 June 2025).

6. Our definition of uncanny comes from Freud. In his essay titled *Das Unheimliche* (i.e. *The Uncanny*; 1919), Freud defines the *uncanny* as not something simply weird but more specifically as something strangely familiar. Following Freud, in his monograph titled *The Uncanny* (2003), Nicholas Royle explains: ‘The uncanny is not simply an experience of strangeness and alienation. More specifically, it is a peculiar commingling of the familiar and unfamiliar. It can take the form of something familiar unexpectedly arising in a strange and unfamiliar context, or of something strange and unfamiliar unexpectedly arising in a familiar context’ (2003: 1). In an article published in 2015, Stuart Jeffrey calls the sense of uncanniness experienced in the digital world a sense of weirdness (145–6). He argues that, for many people, the world of modelled digital objects brings with it a sense of *weirdness*, almost *strangeness* that is especially linked to the fact that these objects have neither substance nor location, as it is not clear to us what physical space such objects actually occupy since there is no degradation and they cannot physically decay, as their original counterparts would have done.

7. https://youtu.be/Jpl-fVBidrE?feature=shared. (Accessed on 2 June 2025)

8. https://www.h2iosc.cnr.it/. (Accessed on 5 March 2025)
